# Six-year trajectories and associated factors of positive and negative symptoms in schizophrenia patients, siblings, and controls: Genetic Risk and Outcome of Psychosis (GROUP) study

**DOI:** 10.1038/s41598-023-36235-9

**Published:** 2023-06-09

**Authors:** Tesfa Dejenie Habtewold, Natalia Tiles-Sar, Edith J. Liemburg, Amrit Kaur Sandhu, Md Atiqul Islam, H. Marike Boezen, GROUP Investigators, Behrooz Z. Alizadeh, Therese van Amelsvoort, Agna A. Bartels-Velthuis, Lieuwe de Haan, Frederike Schirmbeck, Claudia J. P. Simons, Jim van Os, Richard Bruggeman, Behrooz Z. Alizadeh

**Affiliations:** 1 Department of Epidemiology, University Medical Center Groningen, University of Groningen, Hanzeplein 1, 9713 GZ Groningen, The Netherlands; 2 Rob Giel Research Center, University Medical Center Groningen, University Center for Psychiatry, University of Groningen, Groningen, The Netherlands; 3 Department of Statistics, Jagannath University, Dhaka, 1100 Bangladesh; 4 Department of Clinical and Developmental Neuropsychology, Faculty of Behavioral and Social Sciences, University of Groningen, Groningen, The Netherlands; 5 Department of Psychiatry and Neuropsychology, School for Mental Health and Neuroscience, Maastricht University Medical Center, Maastricht, The Netherlands; 6 Department of Psychiatry, Amsterdam UMC, University of Amsterdam, Amsterdam, The Netherlands; 7 Arkin, Institute for Mental Health, Amsterdam, The Netherlands; 8 GGzE Institute for Mental Health Care, Eindhoven, The Netherlands; 9 Department of Psychiatry, Brain Centre Rudolf Magnus, University Medical Center Utrecht, Utrecht University, Utrecht, The Netherlands; 10 Department of Psychosis Studies, Institute of Psychiatry, King’s College London, King’s Health Partners, London, UK

**Keywords:** Epidemiology, Genetics research, Risk factors, Predictive markers, Genetic markers, Schizophrenia

## Abstract

Positive and negative symptoms are prominent but heterogeneous characteristics of schizophrenia spectrum disorder (SSD). Within the framework of the Genetic Risk and Outcome of Psychosis (GROUP) longitudinal cohort study, we aimed to distinguish and identify the genetic and non-genetics predictors of homogenous subgroups of the long-term course of positive and negative symptoms in SSD patients (n = 1119) and their unaffected siblings (n = 1059) in comparison to controls (n = 586). Data were collected at baseline, and after 3- and 6-year follow-ups. Group-based trajectory modeling was applied to identify latent subgroups using positive and negative symptoms or schizotypy scores. A multinomial random-effects logistic regression model was used to identify predictors of latent subgroups. Patients had decreasing, increasing, and relapsing symptoms course. Unaffected siblings and healthy controls had three to four subgroups characterized by stable, decreasing, or increasing schizotypy. PRS_SCZ_ did not predict the latent subgroups. Baseline symptoms severity in patients, premorbid adjustment, depressive symptoms, and quality of life in siblings predicted long-term trajectories while were nonsignificant in controls. In conclusion, up to four homogenous latent subgroups of symptom course can be distinguished within patients, siblings, and controls, while non-genetic factors are the main factors associated with the latent subgroups.

## Introduction

Schizophrenia spectrum disorders are a highly heterogeneous disorder manifested by positive (i.e. delusions, hallucinations, disorganized thinking, speech, and motor behavior) and negative (i.e. diminished emotional expression, avolition, alogia, anhedonia, and asociality) symptoms^1^. Schizophrenia is a severe and chronic form of schizophrenia spectrum disorders and is often followed by poor clinical and functional outcomes^2^. Both positive and negative symptoms are crucial determinants of patients’ recovery, along with cognitive deficits, other clinical (e.g., depressive symptoms, comorbidity), and non-clinical (e.g., life skills, social support, stigma) characteristics^3–7^. Positive symptoms often respond to antipsychotic treatment, whereas negative symptoms are more complex to understand and tackle^2,3^. Negative symptoms can be primary (intrinsic to schizophrenia) and secondary (caused by other factors such as positive symptoms, medication side-effects, substance abuse, and social isolation) but current research and clinical practice target them as one class of undistinguishable negative symptoms. Persistent negative symptoms are associated with poor clinical outcomes and resistance to antipsychotic treatment, including clozapine^8^. According to the guidance of the European Psychiatry Association on the treatment of negative symptoms, treatment with second-generation antipsychotics (e.g., cariprazine), antidepressants, psychosocial rehabilitation (e.g., social skills training), cognitive remediation, and exercise have shown beneficial effects on negative symptoms^9^. Meanwhile, the psychopathologic mechanisms that underpin negative symptoms remain poorly understood compared to positive symptoms^10^.

The pathogenic mechanisms of schizophrenia remain largely unknown despite advances in technology that facilitate biological inquiry to disentangle the molecular complexity^11^. Positive and negative symptoms may share part of their etiopathogenic mechanisms, they may invoke each other, or symptom-specific mechanisms may contribute to their presentations^12–14^. While positive symptoms follow a pattern of reduction and stabilization over time, negative symptoms often present a persistent course over time^15,16^. On the other hand, a study showed that these symptoms follow parallel trajectories over time and are positively associated with each other^12^.

To understand the wide difference in disease presentation, course, and molecular mechanisms, subphenotyping study is recommended. Subphenotyping (i.e., targeting pattern of symptoms, which may be physiologically distinct and have different genetic causes)^17^ has been conducted since the beginnings of modern psychiatry during the 1970’s and has been present throughout its history using both hypothesis-driven and statistical approaches. Recently, some studies have used subphenotyping to examine the heterogeneity of schizophrenia symptoms^18–20^. Compared to other earlier editions, the fifth edition of Diagnostic and Statistical Manual of Mental Disorders (DSM-5) also emphasizes a dimensional/subphenotyping approach^1^. Moreover, cross-sectional, and longitudinal data-driven studies have observed heterogeneity within symptoms in a specific study population (e.g., patients, siblings, controls) and identified several sociodemographic, cardiometabolic clinical factors that distinguish symptoms level among homogeneous subgroups of individuals^21,22^.

The phenotypic heterogeneity and molecular complexity of schizophrenia can be attributed to genetic, sociodemographic, functional, and clinical (disease-related) factors. Our previous review showed that symptom subtypes identified in cross-sectional and longitudinal studies were consistently linked with age, gender, ethnicity, age of illness onset, diagnosis, duration of untreated psychosis, duration of illness, premorbid adjustment, global functioning and quality of life, and cognitive performance^23^. Genetic aetiology of symptoms in schizophrenia is also supported by candidate gene and polygenic risk score (PRS) association studies^24^. Findings from the Psychiatric Genomics Consortium (PGC) genome-wide association study (GWAS) provide convincing evidence for an association of genetic variants with positive and negative symptoms. Moreover, the polygenic risk score for schizophrenia (PRS_SCZ_), which is a measure of cumulative genetic risk, has been associated with positive and negative symptoms in patients with schizophrenia and the healthy population, though large inconsistencies have been observed^18–20^.

Despite the advantages of subphenotyping and group-based trajectory modeling or clustering, only a few studies with a small sample size examined homogeneous subgroups of individuals based on positive and negative symptoms level in patients and siblings while comparing them to those of healthy people. Also, the identified predictors are often only based on pair-wise comparisons and univariable regression. So that, adjustment to confounders was mostly neglected, and the reported effect estimates may have been confounded and biased. Of interest, the role of PRS_SCZ_ to predict positive and the severity of negative symptoms among homogeneous subgroups has not been investigated using data-driven approaches^18,25^. Mostly, cross-sectional studies with small sample size examined the association between PRS_SCZ_ and positive and negative symptoms by using analyses model that adjusted to no or limited confounding factors^18,20,25,26^.

In this study, we combined subphenotyping, polygenic risk scoring and data-driven approaches to strengthen earlier efforts for tackling clinical heterogeneity of schizophrenia spectrum disorders. Therefore, we aimed to investigate latent subgroups using positive and negative symptoms in patients, siblings, and controls. We also examined the association of non-genetic (sociodemographic, functional, cardiometabolic and clinical) and genetic (PRS_SCZ_) factors with identified latent subclasses of the longitudinal course of positive and negative symptoms. Unaffected relatives and controls were included as a comparison group for symptoms severity level and trajectories.

## Results

### Baseline characteristics of participants

Three-fourths of patients were male (p < 0.001) and patients were younger than controls (p < 0.001). Participants significantly differed in PRS_SCZ_ and sociodemographic, functional, and clinical characteristics at baseline (Table 1). The mean PRS_SCZ_ at P_T_ of 0.05, 0.1, and 0.5 of patients was significantly higher than that of siblings and controls (p < 0.001) and the mean PRS_SCZ_ of siblings was significantly higher than that of controls (p < 0.001). In general, characteristics of unaffected siblings laid between patients and controls.

**Table 1:** Participants baseline characteristics.

Variable	Participants			Overall group difference	Pair-wise comparisons
	Controls (C)	Siblings (S)	Patients (P)		
Background characteristics					
Age, mean (SE)	30.60 (0.39)	27.70 (0.28)	27.40 (0.27)	F = 24.0, p < 0.001	P < C; S < C
Gender, male n (%)	269 (46.1)	477 (45.6)	857 (75.4)	X^2^ = 240.97, p < 0.001	
Ethnicity, Caucasian n (%)	523 (92.1)	871 (83.6)	871 (79.1)	X^2^ = 45.84, p < 0.001	
Years of education, mean (SE)	15.6 (0.16)	13.5 (0.12)	12.4 (0.12)	F = 64.5, p < 0.001	P < S & C; S < C
Marital status n (%)					
Not married	319 (57.6)	589 (57.5)	960 (87.8)	X^2^ = 303.17, p < 0.001	
Married/Living together	219 (39.5)	412 (40.2)	104 (9.5)		
Other (divorced and widowed)	16 (2.9)	24 (2.3)	30 (2.7)		
Estimated current IQ, mean (SE)	109.61 (0.69)	102.58 (0.52)	94.81 (0.49)	F = 190.93, p < 0.001	P < S & C; S < C
Premorbid adjustment, mean (SE)	1.13 (0.03)	1.11 (0.02)	1.98 (0.02)	F = 448.7, p < 0.001	P > S; P > C
Age onset illness, mean (SE)			23.1 (0.23)		
Duration of illness, mean (SE)			4.98 (4.46)		
Number of psychotic episodes, mean (SE)			1.72 (1.17)		
Use of antipsychotics^a^ n (%)					
Not currently using			38 (5.22)		
Currently using			574 (78.85)		
Unknown if currently using			116 (15.93)		
Type of antipsychotics n (%)					
Typical			80 (10)		
Atypical			717 (90)		
Cannabis use	83 (14.4)	192 (18.6)	424 (38.2)	X^2^ = 156.80, p < 0.001	
Polygenic risk score of SCZ, mean (SE)					
PRS_SCZ_ (P_T_ = 5 × 10^–8^)	− 0.54 (0.69)	− 0.39 (0.64)	− 0.29 (0.69)	F = 16.40, p = 0.001	P > C
PRS_SCZ_ (P_T_ = 0.05)	− 453.72 (4.75)	− 452.21 (4.61)	− 450.16 (4.77)	F = 75.07, p < 0.001	P > S & C; S > C
PRS_SCZ_ (P_T_ = 0.1)	− 586.57 (5.50)	− 584.94 (5.25)	− 582.49 (5.49)	F = 75.70, p < 0.001	P > S & C; S > C
PRS_SCZ_ (P_T_ = 0.5)	− 902.40 (6.70)	− 900.58 (6.43)	− 897.62 (6.79)	F = 70.04, p < 0.001	P > S & C; S > C
Schizotypy, mean (SE)					
Positive	0.30 (0.02)	0.38 (0.01)		F = 59.45, p < 0.001	S > C
Negative	0.23 (0.01)	0.27 (0.0.01)		F = 49.25, p < 0.001	S > C
Psychotic symptoms, mean (SE)					
Positive			13.90 (0.20)		
Negative			14.99 (0.21)		
Disorganization			16.77 (0.20)		
Emotional distress			15.82 (0.18)		
Excitement			12.05 (0.13)		
Psychotic experiences, mean (SE)					
Positive symptoms frequency	0.19 (0.01)	0.20 (0.01)	0.67 (0.01)	F = 571.97, p < 0.001	P > S & C
Positive symptoms distress	0.43 (0.03)	0.46 (0.02)	1.25 (0.02)	F = 511.36, p < 0.001	P > S & C
Negative symptoms frequency	0.49 (0.02)	0.54 (0.01)	1.02 (0.01)	F = 401.58, p < 0.001	P > S & C
Negative symptoms distress	0.67 (0.02)	0.68 (0.02)	1.25 (0.02)	F = 305.75, p < 0.001	P > S & C
Depressive symptoms frequency^a^	0.58 (0.02)	0.62 (0.02)	1.00 (0.02)	F = 217.76, p < 0.001	P > S & C
Depressive symptoms distress^a^	0.88 (0.03)	0.92 (0.02)	1.44 (0.02)	F = 212.19, p < 0.001	P > S & C
General cognition	0.62 (0.07)	0.13 (0.05)	− 1.18 (0.05)	F = 312.77, p < 0.001	P < S & C; S < C
Functioning and quality of life, mean (SE)					
Occupational functioning^a^	8.93 (0.13)	8.92 (0.10)	5.91 (0.10)	F = 292.98, p < 0.0001	P < S & C
Social functioning^a^	124.04 (0.36)	122.32 (0.28)	112.51 (0.27)	F = 474.62, p < 0.0001	P < S & C; S < C
Global functioning			54.50 (0.52)		
Quality of life	4.07 (0.02)	3.97 (0.02)	3.40 (0.02)	F = 492.16, p < 0.0001	P < S & C; S < C

*SE* standard error.

^a^Used from the second wave at three-year follow-up.

### Trajectories of positive and negative symptoms

**Figure 1:**
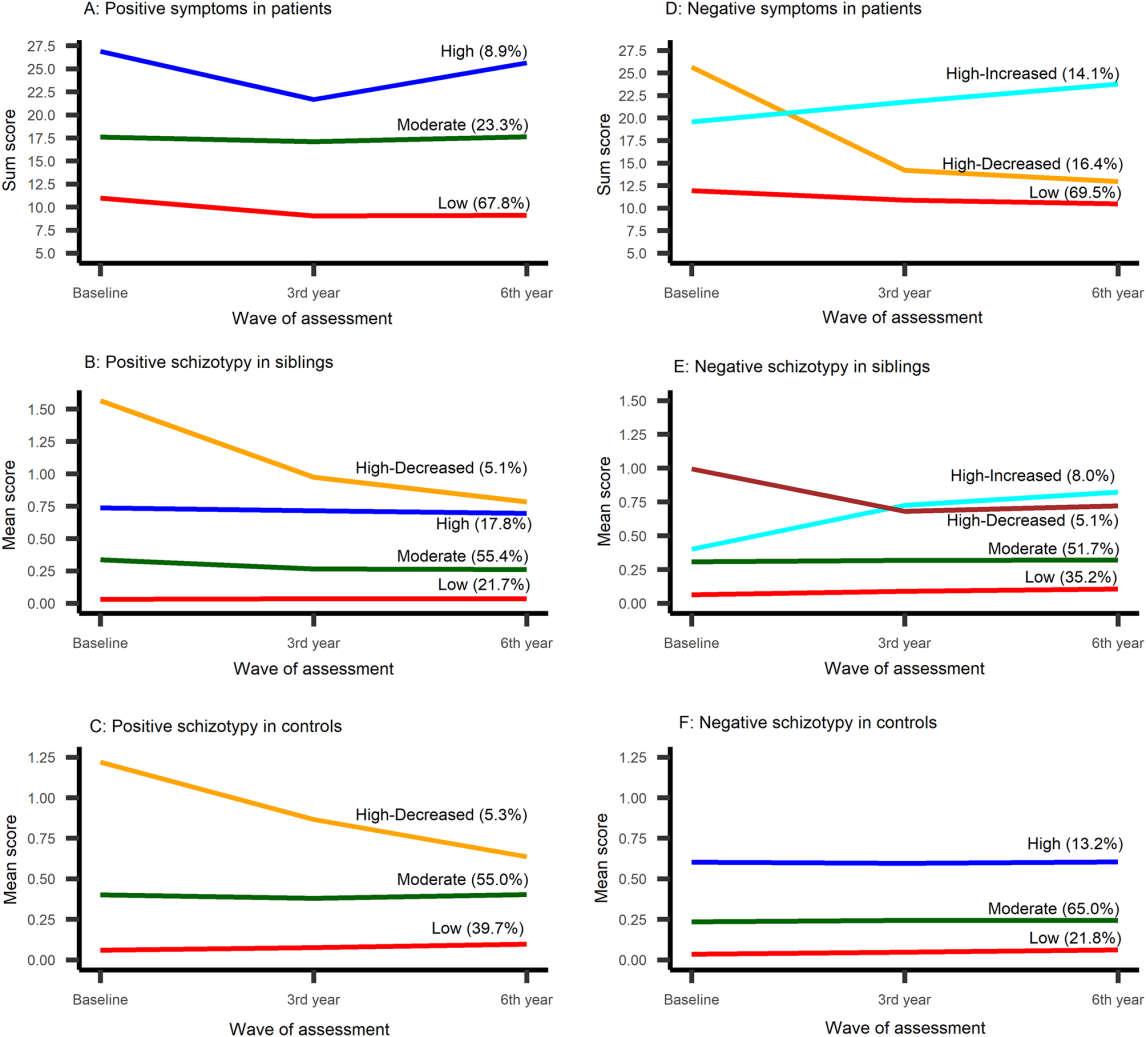
Trajectories of positive and negative symptoms in patients, siblings, and controls.

As Fig. 1 shows, multiple clinically and statistically meaningful trajectories of positive and negative symptoms were identified across all samples. In 1136 eligible patients, wedistinguished three trajectories of positive symptoms that were labeled as low, moderate, and high and three trajectories of negative symptoms, labeled as low, high-decreased, and high-increased. In sensitivity analysis, the core negative symptoms trajectories (data not shown) were highly similar to the original in terms of shapes and frequencies. Chi-square showed very low probability that these distributions are independent (Chi-squared = 1353.3, df = 4, p-value < 2.2e−16) as the vast majority of patients were allocated in the same trajectory shape in both analyses. Only 9% of patients were allocated to different groups in these two analyses (over the total PANSS score and over the core symptoms), while 3% got allocated to the opposite group.

In 1045 eligible siblings, we found four trajectories of positive symptoms: low, moderate, high, and high-decreased and four trajectories of negative symptoms low, moderate, high-decreased and high-increased. In 583 controls, we found three trajectories of positive symptoms: low, moderate, and high-decreased, and three stable trajectories of negative symptoms such as low, moderate, and high. The model accuracy in all group modeling ranged from 70 to 91%. In general, larger variation in trajectories was observed in patients with stability and persistence of symptoms in about two-thirds of patients, reduction of symptoms in more than one-fifth of patients, and worsening symptoms in about one-tenth of patients. Trajectory model fit indices and parameter estimates are presented in Supplementary Tables S1–S8.

### Predictors of positive and negative symptoms trajectories

In patients, univariable regression analysis showed that various sociodemographic, functional, cardiometabolic, and clinical factors were significantly associated with positive and negative symptom trajectories in patients (Supplementary Table S9). PRS_SCZ_ (at all p-value thresholds tested) was not significantly associated with positive and negative symptoms trajectory (Supplementary Table S9). Following adjustment for covariables, only baseline positive and negative symptoms were significantly associated with positive (High vs Low: OR 2.77; 95% CI 1.46–5.26; p = 0.002) and negative symptom trajectories (High-Decreased vs Low: OR 2.15; 95% CI 1.78–2.60; p < 0.0001; and High-Increased vs Low: OR 1.60; 95% CI 1.38–1.86; p < 0.0001) respectively (Table 2). We found similar results in the sensitivity analysis using only core negative symptoms (High-Decreased vs Low: OR 2.08; 95% CI 1.14–3.77; p = 0.016).

**Table 2:** Multivariable multinomial random-effects logistic regression model on the predictors of positive and negative symptoms trajectories in patients.

Predictors	Positive symptom trajectories (Ref = Low)				Negative symptom trajectories (Ref = Low)			
	Moderate		High		High-Decreased		High-Increased	
	OR (95% CI)	P-value	OR (95% CI)	P-value	OR (95% CI)	P-value	OR (95% CI)	P-value
Schizophrenia polygenic risk scores								
PRS_PT=0.05_	1.04 (0.91; 1.18)	0.58	0.94 (0.75; 1.19)	0.62	0.91 (0.81; 1.02)	0.12	0.94 (0.85; 1.03)	0.19
Demographic and clinical characteristics								
Gender, female	0.88 (0.32; 2.44)	0.81	0.12 (0.01; 1.86)	0.13	1.08 (0.29; 4)	0.90	0.97 (0.31; 3.03)	0.95
Ethnicity, non-Caucasian	1.02 (0.23; 4.56)	0.98	9.30 (0.48; 178.47)	0.14				
Years of education (full-time)					0.98 (0.85; 1.13)	0.77	0.99 (0.87; 1.12)	0.88
Duration of illness	0.93 (0.80; 1.08)	0.35	1.11 (0.86; 1.43)	0.44				
Psychotic episode	1.12 (0.72; 1.73)	0.62	1.70 (0.75; 3.83)	0.20				
Premorbid adjustment	1.43 (0.69; 2.97)	0.34	1.19 (0.35; 4.07)	0.78	1.00 (0.52; 1.91)	0.99	1.30 (0.76; 2.21)	0.33
Premorbid IQ	0.99 (0.94; 1.04)	0.62	1.02 (0.93; 1.12)	0.73	1.02 (0.96; 1.08)	0.59	1.03 (0.97; 1.08)	0.36
Neurocognitive function	0.84 (0.51; 1.39)	0.50	0.47 (0.18; 1.22)	0.12	0.79 (0.45; 1.36)	0.39	0.72 (0.45; 1.16)	0.17
Psychotic symptoms								
Positive symptoms	**1.40 (0.80; 2.44)**	**0.23**	**2.77 (1.46; 5.26)**	**0.002**	1.02 (0.91; 1.15)	0.71	1.07 (0.96; 1.18)	0.21
Negative symptoms	1.01 (0.92; 1.10)	0.91	1.11 (0.95; 1.30)	0.18	**2.15 (1.78; 2.6)**	** < 0.0001**	**1.60 (1.38; 1.86)**	** < 0.0001**
Disorganization	1.01 (0.92; 1.12)	0.80	0.92 (0.74; 1.14)	0.45	0.93 (0.83; 1.03)	0.17	1.01 (0.92; 1.11)	0.87
Emotional distress	0.99 (0.88; 1.11)	0.81	1.04 (0.83; 1.32)	0.72	0.99 (0.86; 1.13)	0.83	0.94 (0.83; 1.06)	0.34
Excitement	1.01 (0.85; 1.2)	0.88	0.96 (0.72; 1.28)	0.77	1.14 (0.94; 1.40)	0.19	1.02 (0.86; 1.22)	0.82
Quality of life and functioning								
Health-related Quality of life	0.52 (0.15; 1.8)	0.30	2.61 (0.27; 24.81)	0.40	0.34 (0.10; 1.15)	0.08	**0.26 (0.09; 0.75)**	**0.01**
Global functioning	1.01 (0.97; 1.05)	0.64	1.00 (0.92; 1.08)	0.99	0.99 (0.95; 1.04)	0.76	0.99 (0.96; 1.03)	0.75

Significant values are in bold.

In siblings, several sociodemographic, functional, and clinical factors were significantly associated with positive and negative symptom trajectories (Supplementary Table S10). Additionally, PRS_SCZ_ (at p-value thresholds 0.05 and 0.1) was significantly associated with positive symptom trajectories (High vs Low: OR 1.09; 95% CI 1.03–1.15, p = 0.003) (Supplementary Table S10). In the multivariable model, only quality of life (High vs Low: OR 0.27; 95% CI 0.11–0.68; p = 0.01) was found to be a strong predictor of positive symptoms trajectory, whereas premorbid adjustment (Moderate vs Low: OR 2.02; 95% CI 1.11–3.68; p = 0.02; High vs Low: OR 4.28; 95% CI 2.05–8.95; p = 0.0001) and quality of life (Moderate vs Low: OR 0.16; 95% CI 0.05–0.49; p = 0.0015; High vs Low: OR 0.06; 95% CI 0.01–0.21; p < 0.0001) were found to be a strong predictor of negative symptoms trajectory (Table 3).

**Table 3:** Multivariable multinomial random-effects logistic regression model on the predictors of positive and negative symptom trajectories in siblings.

Predictors	Positive symptom trajectories (Ref = Low)				Negative symptom trajectories (Ref = Low)			
	Moderate		High (high and high-decreased)		Moderate		High (increased and decreased)	
	OR (95% CI)	P-value	OR (95% CI)	P-value	OR (95% CI)	P-value	OR (95% CI)	P-value
Schizophrenia polygenic risk scores								
PRS_PT=0.05_	1.01 (0.95; 1.08)	0.66	1.08 (1.00; 1.17)	0.05	1.00 (0.93; 1.07)	0.92	1.01 (0.91; 1.12)	0.84
Demographic and clinical characteristics								
Age	0.99 (0.96; 1.02)	0.50	0.98 (0.93; 1.02)	0.30				
Gender, female	1.46 (0.82; 2.58)	0.20	0.66 (0.32; 1.34)	0.25				
Marital status, married					0.82 (0.45; 1.49)	0.51	0.73 (0.30; 1.78)	0.49
Years of education (full-time)	0.96 (0.89; 1.03)	0.24	0.96 (0.88; 1.05)	0.34				
Premorbid adjustment	1.41 (0.89; 2.24)	0.14	1.09 (0.63; 1.89)	0.75	**2.02 (1.11; 3.68)**	**0.02**	**4.28 (2.05; 8.95)**	**0.0001**
Premorbid IQ	1.01 (0.99; 1.03)	0.32	1.00 (0.97; 1.02)	0.75				
Psychotic-like experiences (distress)								
Positive symptoms	0.70 (0.34; 1.43)	0.33	1.16 (0.51; 2.65)	0.72	1.62 (0.70; 3.78)	0.26	**3.00 (1.04; 8.68)**	**0.04**
Negative symptoms	2.22 (0.91; 5.43)	0.08	2.41 (0.85; 6.81)	0.10	1.34 (0.52; 3.47)	0.55	2.45 (0.66; 9.05)	0.18
Depressive symptoms	0.86 (0.43; 1.74)	0.68	**2.67 (1.13; 6.32)**	0.03	2.05 (0.89; 4.70)	0.09	1.89 (0.59; 6.06)	0.28
Quality of life and functioning								
Health-related quality of life	**0.58 (0.28; 1.24)**	**0.16**	**0.27 (0.11; 0.68)**	**0.01**	**0.16 (0.05; 0.49)**	**0.0015**	**0.06 (0.01; 0.21)**	** < 0.0001**

Significant values are in bold.

In controls, positive and negative symptom trajectories were significantly associated with diverse sociodemographic, functional, and clinical factors (Supplementary Table S11). PRS_SCZ_ (at all p-value thresholds tested) was not significantly associated with positive and negative symptoms trajectory (Supplementary Table S11). In the multivariable model, age (High-decreased vs Low: OR 0.91, 95% CI 0.83–0.99, p = 0.04) and quality of life (High-decreased vs Low: OR 0.04; 95% CI 0.002–0.76; p = 0.03) was found to be a strong predictor of positive symptoms trajectory, whereas premorbid adjustment (High vs Low: OR 7.74; 95% CI 1.01–59.38; p = 0.05) were found to be a strong predictor of negative symptoms trajectory (Table 4).

**Table 4:** Multivariable multinomial random-effects logistic regression model on the predictors of positive and negative symptom trajectories in controls.

Predictors	Positive symptom trajectories (Ref = Low)				Negative symptom trajectories (Ref = Low)			
	Moderate		High-decreased		Moderate		High	
	OR (95% CI)	P-value	OR (95% CI)	P-value	OR (95% CI)	P-value	OR (95% CI)	P-value
Schizophrenia polygenic risk scores								
PRS_PT=0.05_	1.03 (0.90; 1.19)	0.64	0.96 (0.8; 1.16)	0.69	1.01 (0.90; 1.13)	0.87	1.00 (0.88; 1.14)	0.97
Demographic and clinical characteristics								
Age	0.96 (0.89; 1.02)	0.20	0.91 (0.83; 1.00)	0.05				
Ethnicity, non-Caucasian	2.01 (0.14; 28.05)	0.60	0.84 (0.02; 37.89)	0.93				
Premorbid adjustment	2.68 (0.58; 12.34)	0.20	2.50 (0.4; 15.63)	0.33	2.01 (0.49; 8.24)	0.33	**5.10 (1.1; 23.57)**	**0.04**
Psychotic-like experiences (distress)								
Positive symptoms	1.42 (0.26; 7.68)	0.68	3.25 (0.37; 28.45)	0.29	2.40 (0.52; 11.07)	0.26	5.39 (0.89; 32.75)	0.07
Negative symptoms	2.62 (0.36; 19.27)	0.34	2.47 (0.16; 38.45)	0.52	0.62 (0.13; 2.95)	0.55	0.21 (0.03; 1.65)	0.14
Depressive symptoms	2.75 (0.44; 17.30)	0.28	4.55 (0.44; 47.14)	0.20	4.65 (0.39; 54.66)	0.22	8.48 (0.62; 116.37)	0.11
Quality of life and functioning								
Health-related quality of life	0.19 (0.02; 2.20)	0.18	**0.04 (0.00; 0.79)**	**0.03**	0.60 (0.09; 3.85)	0.59	**0.10 (0.01; 0.86)**	**0.04**

Significant values are in bold.

## Discussion

We investigated the long-term trajectories of positive and negative symptoms among patients with schizophrenia-spectrum disorder, their unaffected siblings, and healthy controls. We also examined the role of genetic (PRS_SCZ_), sociodemographic, functional, cardiometabolic, and clinical factors.

In this study, three to four subgroups of patients, siblings, and controls were identified based on positive and negative symptoms. This is in line with previous studies, as presented in our recent systematic review, that identified two to five latent groups across psychotic symptom dimensions and population groups (i.e., patients, siblings and controls), though the study characteristics and findings (e.g., actual number identified subgroups and percentage distribution in each subgroup) were different for individual studies. The use of different statistical modeling techniques may lead to the identification of a different number of subgroups. For example, a study on the application of five statistical data-driven subtyping methods on longitudinal data showed that the number of trajectory groups derived from one method can be remarkably different from the other method using the same data structure^27^. Differences in instruments to measure clinical symptom severity may also affect the number of identified trajectories.

We found a significant association between PRS_SCZ_ and the six-year trajectory of positive symptoms in patients and siblings but attenuated after adjustment for sociodemographic, clinical, and functional factors. Earlier studies on the association between schizophrenia phenotypes and PRS_SCZ_ have largely shown inconsistent results, and some are concordant with our findings from the bivariate correlation analyses. A systematic review of PRS-based studies showed that PRS_SCZ_ was significantly associated with the severity of negative symptoms, but not with positive symptoms, in patients and the healthy general population wherein most estimates were based on correlation tests^26^. A population-based birth cohort study showed that PRS_SCZ_ was significantly associated with negative symptoms in adolescence in the general population, while others found no evidence for an association between PRS_SCZ_ and psychotic experiences/positive symptoms^20,28^. Other cross-sectional and longitudinal studies^18,25,29,30^ found no association between PRS_SCZ_ and positive and/or negative symptoms in patients and healthy individuals. The inconsistencies between studies could be due to the difference in the assessment instruments for positive and negative symptoms, sampling, sample size, genotyping, and quality control methods used in GWAS, and inclusion of patients at vastly different stages of illness and with a diverse spectrum of symptoms. Additionally, PRS is highly dependent on factors, such as the sample characteristics, sample size, stage, and/or severity of the disease that leads to variation in findings across studies^31^. Furthermore, the PRS was derived from a schizophrenia diagnosis, which is expected to represent less than half the participants with negative symptoms due to the schizophrenia illness itself (i.e. primary negative symptoms) and may not be predictive for negative symptoms related to secondary causes (i.e. secondary negative symptoms), such as substance abuse and depression.

In line with an earlier study^15^, baseline positive and negative symptoms significantly predicted positive and negative symptom trajectories in patients, respectively. This finding suggests that patients who had severe symptoms at baseline showed persistence and stability of the initial level of symptoms^32^. On the other hand, an earlier study showed that the severity of positive symptoms initially decreased and became stable over time while negative symptoms showed persistence over time^16^. In siblings, poor premorbid adjustment and quality of life were found to be strong predictors of positive and negative trajectories. In controls, none of the environmental factors survived adjustment for covariables. The lack of association following adjustment for covariables in our study is not surprising given that these factors were selected from previous group-based trajectory modeling studies that mostly performed univariable analyses or just compared trajectories using proportion or mean estimates. In general, at least in the univariable model, multiple factors were found to be strong predictors of positive and negative symptoms trajectories, and this may support the notion that positive and negative symptoms share a similar course^12–14^.

We investigated the long-term trajectories of schizophrenia symptoms and their association with a broad range of sociodemographic, clinical, functional, and genetic (PRS_SCZ_) factors in family-based cohorts with large samples of people with psychosis, their unaffected relatives, and controls. This eases the comparison of the patterns and offers new perspective on genetic and environmental contributions to the development of trajectories. Interestingly, results on the number of the identified latent subgroups and course over time were comparable across patients, unaffected siblings and healthy controls, and provided a similar trend of evidence, though the predictors and level of significance were different. Furthermore, we ensured the validity and clinical meaningfulness of the identified trajectories by observing heterogeneity across all sample groups, comparing findings from previous studies in our systematic review, and involving clinicians during trajectory modeling. However, the current study has also some limitations. PRS is based on common genetic variants and naturally does not capture copy number variants (CNV), or rare SNP contributions to variance. Besides, trajectory group identification and their association with PRS_SCZ_ may be different if it is done based on other symptom definitions, e.g. total PANSS score, a subdomain of positive (e.g., hallucination, delusion, disorganization), or PRS constructed based GWAS summary statistics of these quantitative phenotypes. Another limitation is related to the use of PANSS to assess negative symptoms. This scale includes aspects that are not conceptualized as negative symptoms, but it evaluates symptoms belonging to the experiential domain (avolition, anhedonia, asociality) only at the behavioral level. There is also no distinction between primary and secondary symptoms in our study, which could have quite different trajectories. Extrapyramidal side effects impact negative symptoms; unfortunately, they were not measured in GROUP and hence were not accounted for. Regarding PANSS positive symptoms, reality distortion (e.g., hallucinations and delusions) is quite distinct from the disorganization of thought. Moreover, the trajectory analyses were not cross-validated in an independent sample or in split-sample.

## Conclusions

Three to four trajectories or latent subgroups were identified in patients, relatives, and controls. PRS_SCZ_ did not predict latent subgroups and long-term trajectories of positive and negative symptoms in patients, siblings, and controls. Among non-genetic factors, baseline symptoms severity in patients, and premorbid adjustment, and health-related quality of life in siblings predicted long-term trajectories while none of them were significant in controls. Added to previous knowledge, the longitudinal clinical course of schizophrenia can be distinguished into predictable stable trajectories when non-genetic factors may be sufficient to distinguish latent subgroups and predict their longitudinal course of schizophrenia symptoms. A prior prediction of the best fit corresponding clinical trajectory would guide psychiatrists for choosing of the right patient-tailored intervention when treating first episode schizophrenia. Large-scale longitudinal studies with robust measures of quantitative phenotypes using a harmonized measuring instrument are needed to determine how genetic risk for schizophrenia is expressed and whether this expression changes with time, to examine potential mediators and moderators of risk, and to determine the usefulness of PRS_SCZ_ for prediction of transition to psychosis in siblings and healthy people.

## Methods

### Study population

Data of a 6-year longitudinal multi-center national study was analyzed comprising 1119 patients, 1059 unaffected siblings, and 586 healthy controls who were eligible at baseline, using the 7th official release of the Genetic Risk and OUtcome of Psychosis (GROUP) cohort data. Patients were included if diagnosed with a schizophrenia spectrum disorder, age range of 16 to 50 years, good command of the Dutch language, and willing and capable of giving written informed consent. Siblings and controls were included if they had no known lifetime psychotic disorder. The Diagnostic and Statistical Manual of Mental Disorders, Fourth Edition (DSM‐IV) criteria were used to diagnose schizophrenia spectrum disorder with Comprehensive Assessment of Symptoms and History interview^33^. During the 6 years follow-up, 14 siblings and three controls developed psychosis; therefore, they were included in the patient group in all analyses. Sociodemographic, functional, cardiometabolic, clinical and genetic data were collected at baseline, and after 3 years and 6 years. The study was approved by the Ethics Committees of the Ethical Review Board of the University Medical Centre Utrecht and subsequently by local review boards of each participating institute, in accordance with the Declaration of Helsinki. Besides, all methods were performed in accordance with the published study protocol^34^. Details on the sample size and power calculation, sample characteristics, and recruitment and assessment procedures presented in the published protocol^34^.

### Measurement of variables

#### Positive and negative symptoms

The Positive and Negative Syndrome Scale (PANSS) for schizophrenia was administered to measure the severity of positive and negative symptoms in patients^35^. The PANSS is a reliable and valid tool that rates severity of symptoms in an incremental seven-point Likert severity scale (from 1 = none, 2 = minimal, to up to 7 = extreme). The positive symptom subscale score was calculated as the sum of seven positive symptom items, and the negative subscale score was the sum of seven negative symptoms. As part of sensitivity analysis, negative subscale score was also calculated according to recent guidelines on measuring core negative symptoms as a sum of five negative symptoms (excluding previously integrated abstract thinking and stereotyped thinking)^36–38^. Positive and negative schizotypy in siblings and healthy controls were assessed with the Structured Interview for schizotypy‐revised (SIS‐R)^39–41^. The SIS‐R is a reliable tool and items are rated on an incremental four-point Likert severity scale (from 0 = absent, 1 = mild, 2 = moderate, and 3 = severe). The positive dimension score was calculated as the mean of positive schizotypy (referential thinking, delusional mood, magical ideation, illusions, and suspiciousness), and the negative subscale score was calculated as the mean of negative schizotypy (social isolation, social anxiety, introversion, and restricted affect).

#### Sociodemographic, functional, and clinical factors

The sociodemographic variables were age, gender, marital status, ethnicity, and educational status (i.e., years of education). Clinical and functional variables were age of onset of illness, duration of illness, number of psychotic episodes, antipsychotic treatment, substance use, premorbid adjustment, estimated IQ, general cognition, psychotic symptoms or psychotic-like experiences, depression, social and global functioning, and quality of life. The variables are discussed below. We also collected cardiometabolic data, such as body mass index, waist circumference, glycated hemoglobin, triglycerides, low-density lipoprotein, high-density lipoprotein, systolic and diastolic blood pressure and pulse rate through physical and laboratory examination only in patients at the third year of follow-up.

Cognitive function was assessed using a comprehensive neuropsychological test battery^27,42,43^ that included the Word Learning Task (i.e. immediate recall and delayed recall), Continuous Performance Test‐HQ (CPT‐HQ) (CPT performance index and CPT variability), WAIS‐III Digit Symbol Substitution Test, WAIS‐III Information, WAIS‐III Calculation, and WAIS‐III Block Design test. A composite score as a measure of general cognition was generated using principal component analysis. Details on the assessment of these tasks, scoring system and calculation of composite score are published elsewhere^34,44^. The Community Assessment of Psychic Experiences (CAPE) was used to assess psychotic experiences (CAPE-42; www.cape42.homestead.com)^45,46^. The Calgary Depression Scale for Schizophrenia (CDSS) was used to assess depression^47^. Premorbid adjustment, which is a measure of the degree of achievement of developmental goals at each of several periods of a subject’’s life before the onset of schizophrenia, was assessed using the premorbid adjustment scale^48^. The World Health Organization Quality of Life‐BREF (WHOQOL‐BREF) questionnaire, which has high construct validity and reliability, was used to assess the quality of life^49^; the Social Functioning Scale (SFS) and Global Assessment of Functioning (GAF) – disability were used to measure social (total score of SFS), occupational (sub-score of SFS) and global functioning^50,51^.

#### Genotyping, quality control (QC), and polygenic risk score (PRS)

Blood samples were obtained at baseline for genotyping. Genotype data for 2,812 individuals was generated on a customized Illumina, IPMCN array with 570,038 single nucleotide polymorphisms (SNPs). This chip contains ~ 250 k common SNPs, 250 K Exome chip variants (rare, exonic, nonsynonymous, MAF < 1%), and ~ 50 K psychiatric-related variants. Quality control (QC) procedures were performed using PLINK v1.9^52^. In total, 2505 individuals and 275,021 SNPs passed QC steps. SNPs were imputed on the Michigan server^53^ using the HRC r1.1 2016 reference panel with European samples after phasing with Eagle v2.3. Finally, PRSs for 2505 samples were calculated using schizophrenia-associated alleles and effect sizes reported in the GWAS summary statistics from the Psychiatric genetics consortium (PGC) 2022^54^ by excluding Dutch subjects while adjusting for population ancestry estimate (i.e., PCA components). Polygenic risk scores for SCZ were derived from a European-ancestry study^1^ and generated by applying a Bayesian framework method that uses continuous shrinkage (cs) on SNP effect sizes. PRS-cs-auto is robust to varying genetic architectures, provides substantial computational advantages, and enables multivariate modeling of local linkage disequilibrium patterns as described in detail elsewhere. PRSs were analyzed for four schizophrenia GWAS p-value thresholds of 5 × 10^–8^, 0.05, 0.1, 0.5 and 1.0. Each PRS separately modeled to compare results and identify the most predictive and discriminant PRS for observed trajectories. See the Supplementary Methods for detailed information. The top three principal components were used to correct genetic ancestry effect.

### Statistical analysis

Differences in patient, sibling and control sociodemographic, clinical and functional characteristics at baseline were explored using a linear mixed-effects model for continuous variables and Pearson’s Chi-square tests for categorical variables. Genetic profile (PRS_SCZ_) was also compared across samples. Since individuals are clustered in families, observations from the same family are correlated. Therefore, we declared family as a random effect to set up a common correlation among all observations from the same family in the regression modeling. Family as a random effect was ignored when the model poorly fitting (i.e., G-matrix was not positive definite). The Maximum Likelihood (ML) method was used to estimate the model parameters and fixed-effects (i.e., type III overall group comparison tests) model results were interpreted. The bivariate association between PRS_SCZ_ and baseline positive and negative symptoms was also examined using the Spearman’s correlations test. Group-based trajectory modeling (GBTM) using PROC TRAJ was applied to identify latent homogeneous subgroups that have a unique symptom trajectory^55^. The modeling process is explained in detail in our earlier publication^44^ and in the Supplementary Methods. A trajectory is defined as a group of individuals who have a symptom profile that is homogenous within the group but significantly heterogeneous between groups^56^. We used the terms trajectory and latent subgroup interchangeably.

The multinomial random-effects logistic regression model was used because symptomatic trajectories had more than two nominal categories and study participants were clustered within a family. Besides, all analyses used full information ML estimation, which uses all data, including partial cases, to arrive at unbiased parameter estimates. Univariable and multivariable multinomial random-effects logistic regression models^57^ using PROC NLMIXED were fitted using symptomatic trajectories as an outcome variable, and PRS_SCZ_ and sociodemographic, functional, clinical and cardiometabolic factors as predictors. PROC NLMIXED maximizes the likelihood function of the multinomial random-effects model by the Adaptive Gaussian quadrature method and Dual Quasi-Newton optimization technique, and therefore, provides stable parameter estimates. PROC LOGISTIC was used to estimate initial parameters (β) that were used in the random-effects logistic regression model and to calculate explained variance (i.e., *Nagelkerke R*^2^). In the multivariate model, all significant variables at alpha level ≤ 0.025 in the univariable model were included. Variables not collected at baseline assessment (i.e., depressive symptoms, social and occupational functioning, and physical health parameters) were not included to avoid data separation points and to increase model convergence. All tests were adjusted for multiple comparisons by Bonferroni correction; therefore, the significant threshold was set to be 0.025 (i.e., 0.05 divided the number of comparisons). We made two comparisons across all samples with one group used as a reference and two subgroups/trajectories were merged in siblings. The PRS_SCZ_ at p-value threshold 0.05 (i.e., considered as the most predictive) was included in the multivariable model, even when its effect was not significant in the univariable model because only we have PRS_SCZ_ to assess genetic susceptibility. All the analyses were done using R 3.6.0 and SAS 9.3 software.

## Supplementary Information

Supplementary Information.

## Data Availability

The data that support the findings of this study are available from the GROUP cohort principal investigators (B.Z.A. and R.B.) on reasonable request. The data are not publicly available due to containing information that could compromise research participant privacy or consent.
