# Supplementary material for: Six-year trajectories and associated factors of positive and negative symptoms in schizophrenia patients, siblings, and controls: Genetic Risk and Outcome of Psychosis (GROUP) study
Source: Sci Rep. 2023 Jun 9;13:9391. doi: 10.1038/s41598-023-36235-9 (PMC10256804; doi:10.1038/s41598-023-36235-9)
Supplement: Supplementary file 1 — Supplementary Information. [file 41598_2023_36235_MOESM1_ESM.docx]

**Supplementary file**

**Supplementary methods**

***Trajectory modelling***

The censored normal drop-out model was used assuming upper and lower censoring of symptom scores and presence of attrition during follow-up.^1^ The drop-out model, which includes a logistic model of drop-out probability per follow-up period, was used to examine the effect of attrition rates on group membership probabilities assuming outcome observations during follow-up intervals depend on preceding responses.^2^ Moreover, missing data were handled by using a full information maximum likelihood model that provides parameter estimates by maximizing the likelihood function of the incomplete data as it produces less biased results compared with other methods.^3^ Initially, the optimum number of trajectory groups was identified using a quadratic polynomial order. The most parsimonious number of trajectory subgroups was determined using Bayesian information criterion (BIC), logged Bayes factor (i.e. 2*ΔBIC) and our clinical judgment. A 10-point difference was taken to favor the lower BIC.^4^ Then, the polynomial order was adjusted until it became significant for all subgroups at the significance level of 0.05. To measure the model accuracy to distinguish individuals, we calculated the average group posterior probability (AvePP). A model with an AvePP greater than 0.7 for all identified groups is believed to be accurate.^5^ A complete explanation of the theoretical concept, function, and application of GBTM is published elsewhere.^6-8^ To achieve our objectives, trajectory analyses were performed per group (i.e., patient, sibling, healthy control) and symptom domain (i.e., positive and negative).

***Genotyping and*** ***quality control***

Genotype data for 2,812 individuals was generated on a customized Illumina, IPMCN array with 570,038 SNPs. This chip contains ~250k common SNPs, 250K Exome chip variants (rare, exomic, nonsynonymous, minor allele frequency (MAF) < 1%), and ~50K psychiatric-related variants. Quality control procedures were performed using PLINK v1.9.^9^ SNPs and samples with call rates below 95% and 98%, respectively, were removed. A strict SNP QC only for subsequent sample quality control steps was conducted. This involved MAF threshold>10% and a Hardy-Weinberg equilibrium (HWE) p-value>1e-05, followed by linkage disequilibrium (LD) based SNP pruning (R^2^<0.2). This resulted in ~58K SNPs to assess sex errors, heterozygosity (F<3 standard deviation (SD)), homozygosity (F>3SD), and relatedness by pairwise identity by descent (IBD) values. Duplicate samples (pihat > 0.8) were removed and remaining pairs were manually checked since this dataset contains family members. After removing failing samples, a regular SNP QC was performed (SNP call rate>98%, HWE p>1e-06, MAF>1%). After MDS clustering with Hapmap Phase 3 individuals to check ethnicity, samples that deviated more than 3 standard deviations from our dataset were removed (n=91). In addition, the first 20 genetic PCs of passed quality-controlled samples were generated using the strict SNP QC list by EIGENSTRAT. Next, strand ambiguous SNPs and duplicate SNPs were removed. Mendelian errors were set to missing followed by another missingness check (2% threshold) for samples (n=8) and SNPs, and SNPs with differential missingness between cases and controls were removed. In total, 2,505 individuals and 275,021 SNPs passed these abovementioned QC steps. SNPs were imputed on the Michigan server^10^ using the HRC r1.1 2016 reference panel with European samples after phasing with Eagle v2.3. Post-imputation QC involved removing SNPs with an Rsq info score<0.3, with a MAF<0.01, SNPs that had a discordant MAF compared to the reference panel, and strand ambiguous AT/CG SNPs and multi-allelic SNPs.

***Polygenic score calculation***

Polygenic risk scores (PRS) for 2,505 samples were calculated using schizophrenia-associated alleles and effect sizes reported in the GWAS summary statistics from the Psychiatric genetics consortium (PGC) 2022^55^, excluding Dutch subjects. Overlapping SNPs between the PGC GWAS (training dataset), 1000 reference Genome (reference dataset), and our dataset (target dataset) were selected. Then 1) insertion or deletion, ambiguous SNPs, 2) SNPs with MAF <0.01 and SNP with imputation quality (R^2^) < 0.8 in both training datasets and target dataset, and 3) SNPs located in complex-LD regions. were excluded, leaving 2,950,238 SNPs. These SNPs were clumped in two rounds using PLINK; round 1 with the default parameters (physical distance threshold 250kb and LD threshold (R^2^) <0.5; round 2 with a physical distance threshold of 5,000kb and LD threshold (R^2^) < 0.2; resulting 194,665 SNPs for PRS calculation. Odds ratios for autosomal SNPs reported in the schizophrenia summary statistics were log-converted to beta values. PRS were calculated using PLINK’s score function for four schizophrenia GWAS p-value thresholds: 5 x 10^-8^, 0.05, 0.1 and 0.5. In the main analysis, the p-value threshold 0.05 was used because it distinguishes trajectories very well and considered as the most predictive in the original GWAS. The other three thresholds were used for sensitivity analysis.

**Supplementary results**

Table S1: Model fit indices for positive symptoms.

| Number of groups | BIC_n_ | ΔBIC_n_ | Logged Bayes factors (2 x ΔBIC_n_) | Evidence against the Ho | Decision |
| --- | --- | --- | --- | --- | --- |
| Patients | | | | | |
| 1 | -8355.09 | 8355.09 | 16710.18 |  |  |
| 2 | -8082.23 | 272.86 | 545.72 |  |  |
| 3 | **-8045.20** | **37.03** | **74.06** | **Very strong** | **Accept** |
| 4 | -8065.16 | -19.96 | -39.92 | Very weak |  |
| Siblings | | | | | |
| 1 | -2219.40 | 2219.40 | 4438.80 |  |  |
| 2 | -2041.87 | 177.53 | 355.06 |  |  |
| 3 | -1989.16 | 52.71 | 105.42 |  |  |
| 4 | **-1984.37** | **4.79** | **9.58** | **Very strong** | **Accept** |
| 5 | -2001.68 | -17.31 | -34.62 | Very weak |  |
| Controls | | | | | |
| 1 | -1137.38 | 1137.38 | 2274.76 |  |  |
| 2 | -1091.89 | 45.49 | 90.98 |  |  |
| 3 | **-1080.36** | **11.53** | **23.06** | **Very strong** | **Accept** |
| 4* | -1067.73 | 12.63 | 25.26 |  |  |

*BIC_n_ = Sample size adjusted Bayesian information criteria; * = Even though the model indices were decreased, the model couldn’t estimate the parameters due to small number of individuals per trajectory groups. Therefore, we stopped clustering individuals above three groups.*

Table S2: Model fit indices for negative symptoms.

| Number of groups | BIC_n_ | ΔBIC_n_ | Logged Bayes factors (2 x ΔBIC_n_) | Evidence against the Ho | Decision |
| --- | --- | --- | --- | --- | --- |
| Patients | | | | | |
| 1 | -8229.67 | 8229.67 | 16459.34 |  |  |
| 2 | -8019.83 | 209.84 | 419.68 |  |  |
| 3 | **-7966.87** | **52.96** | **105.92** | **Very strong** | **Accept** |
| 4 | -7967.22 | -0.35 | -0.70 | Very weak |  |
| Siblings | | | | | |
| 1 | -1277.67 | 1277.67 | 2555.34 |  |  |
| 2 | -1097.47 | 180.20 | 360.40 |  |  |
| 3 | -1052.61 | 44.86 | 89.72 |  |  |
| 4 | **-1046.15** | **6.46** | **12.92** | **Very strong** | **Accept** |
| 5 | -1051.92 | -5.77 | -11.54 | Very weak |  |
| Controls | | | | | |
| 1 | -616.90 | 616.90 | 1233.80 |  |  |
| 2 | -545.85 | 71.05 | 142.10 |  |  |
| 3 | **-540.13** | **5.72** | **11.44** | **Very strong** | **Accept** |
| 4 | -536.71 | 3.42 | 6.84 | Weak |  |
| 5 | -543.78 | -7.07 | -14.14 | Very weak |  |

*BIC_n_ = Sample size adjusted Bayesian information criteria*

**Interpretation of trajectory model parameter estimates (Table S3 – S8)**

The intercept (β_0_) corresponds to the predicted value of mean cognitive performance when time is equal to zero. The linear slope parameter estimate (β_1_) represents the amount of increase/decrease on the predicted mean cognitive performance for each unit (three years in our study) of increase on time (e.g, Baseline to three years). The quadratic slope parameter estimate (β_2_) represents the amount of increase/decrease in the predicted mean cognitive performance for each unit of increase on the squared time. The t-test for the parameter estimates tests whether the value of mean cognitive performance significantly differs from zero when the time is equal to zero.^12^ Sigma represents the amount of variance in the data explained by our model. The drop-out model, which includes a logistic model of drop-out probability per wave of measurement, was used to investigate whether the attrition rates significantly biased group membership probabilities of trajectories.^2^ Zero represents a constant attrition rate, one represents the attrition rate depends on baseline measurement and two represents the attrition rate depends on the baseline and three years measurement point For each group. In our study, we assumed that the drop-out probability depends on the two previous responses (i.e. baseline and three-year observation) for each group.

Trajectory groups were named by consensus considering the group mean score and the nature of change over time. In patients, the ‘Low’ group represents individuals with positive and negative symptoms sum score of < 15 on the PANSS, ‘Moderate’ group represents individuals with a sum score of approximately 15 to 20 and ‘High’ group represents individuals with a sum score of > 20. Based on the change throughout follow-up, we also labeled trajectories as ‘High-Decreased’ – start with high symptoms and ameliorate over time or ‘High-Increased’ – start with high symptoms and worsen over time. In siblings and controls, we followed a similar approach: ‘Low’ with mean score < 0.25 on the SIS-R, ‘Moderate’ with mean score approximately 0.25 to 0.5, and ‘High’ with a mean score of > 0.5. We also named trajectories in siblings and controls as ‘High-Decreased’ or ‘High-Increased’

**Table S3:** **Trajectory model parameter estimates of positive symptoms in patients.**

| Trajectory | Parameter | Estimate | Standard Error | T-value | P-value |
| --- | --- | --- | --- | --- | --- |
| Groups |  |  |  |  |  |
| Low | Intercept | 15.12 | 0.83 | 18.11 | <0.0001 |
|  | Linear | -5.03 | 0.95 | -5.26 | <0.0001 |
|  | **Quadratic** | **1.02** | **0.24** | **4.23** | **<0.0001** |
| Moderate | **Intercept** | **16.93** | **0.37** | **45.14** | **<0.0001** |
| High | Intercept | 39.60 | 2.81 | 14.07 | <0.0001 |
|  | Linear | -17.75 | 3.26 | -5.44 | <0.0001 |
|  | **Quadratic** | **4.34** | **0.83** | **5.24** | **<0.0001** |
| Dropout |  |  |  |  |  |
| Low | Drop0 | -1.79 | 0.48 | -3.71 | 0.0002 |
|  | Drop1 | 0.07 | 0.04 | 1.68 | 0.0936 |
|  | Drop2 | 0.01 | 0.03 | 0.47 | 0.6367 |
| Moderate | Drop0 | -6.98 | 2.60 | -2.68 | 0.0074 |
|  | Drop1 | 0.23 | 0.10 | 2.33 | 0.0196 |
|  | Drop2 | 0.10 | 0.07 | 1.47 | 0.1420 |
| High | Drop0 | -2.66 | 2.73 | -0.98 | 0.3286 |
|  | Drop1 | 0.01 | 0.05 | 0.12 | 0.9074 |
|  | Drop2 | 0.06 | 0.07 | 0.91 | 0.3618 |
| Sigma |  | 4.28 | 0.07 | 56.81 | <0.0001 |
| Group membership | | | | | |
| Low | (%) | 67.78 | 2.23 | 30.43 | <0.0001 |
| Moderate | (%) | 23.26 | 2.17 | 10.69 | <0.0001 |
| High | (%) | 8.95 | 1.34 | 6.67 | <0.0001 |

**Table S4: Trajectory model parameter estimates of positive symptoms in siblings.**

| Trajectory | Parameter | Estimate | Standard Error | T-value | P-value |
| --- | --- | --- | --- | --- | --- |
| Groups |  |  |  |  |  |
| Low | **Intercept** | **-0.22** | **0.05** | **-4.52** | **<0.0001** |
| Moderate | Intercept | 0.30 | 0.03 | 8.85 | <0.0001 |
|  | **Linear** | **-0.03** | **0.01** | **-2.62** | **0.0088** |
| High | **Intercept** | **0.66** | **0.04** | **15.88** | **<0.0001** |
| High-Decreased | Intercept | 2.45 | 0.27 | 9.18 | <0.0001 |
|  | Linear | -1.14 | 0.30 | -3.81 | 0.0001 |
|  | **Quadratic** | **0.19** | **0.07** | **2.57** | **0.0103** |
| Dropout |  |  |  |  |  |
| Low | Drop0 | -1.62 | 0.27 | -6.07 | <0.0001 |
|  | Drop1 | 10.70 | 5.25 | 2.04 | 0.0417 |
|  | Drop2 | 3.77 | 3.04 | 1.24 | 0.2154 |
| Moderate | Drop0 | -3.78 | 0.91 | -4.14 | <0.0001 |
|  | Drop1 | 4.82 | 1.46 | 3.29 | 0.0010 |
|  | Drop2 | 1.31 | 0.96 | 1.37 | 0.1720 |
| High | Drop0 | 645.26 | 288949.63 | 0.002 | 0.9982 |
|  | Drop1 | -1159.89 | 505507.62 | -0.002 | 0.9982 |
|  | Drop2 | -969.16 | 504857.60 | -0.002 | 0.9985 |
| High-Decreased | Drop0 | -3.11 | 3.20 | -0.97 | 0.3323 |
|  | Drop1 | 2.16 | 1.34 | 1.62 | 0.1055 |
|  | Drop2 | -0.28 | 1.64 | -0.17 | 0.8644 |
| Sigma |  | 0.31 | 0.01 | 46.13 | <0.0001 |
| Group membership | | | | | |
| Low | (%) | 21.67 | 3.47 | 6.25 | <0.0001 |
| Moderate | (%) | 55.39 | 3.50 | 15.84 | <0.0001 |
| High | (%) | 17.82 | 2.88 | 6.19 | <0.0001 |
| High-Decreased | (%) | 5.11 | 1.02 | 5.03 | <0.0001 |

**Table S5: Trajectory model parameter estimates of positive symptoms in controls.**

| **Trajectory** | **Parameter** | **Estimate** | **Standard Error** | **T-value** | **P-value** |
| --- | --- | --- | --- | --- | --- |
| **Groups** |  |  |  |  |  |
| Low | **Intercept** | **-0.06** | **0.04** | **-1.57** | **0.1168** |
| Moderate | **Intercept** | **0.36** | **0.02** | **14.66** | **<0.0001** |
| High-Decreased | Intercept | 1.45 | 0.15 | 9.61 | <0.0001 |
|  | **Linear** | **-0.31** | **0.08** | **-4.04** | **0.0001** |
| **Dropout** |  |  |  |  |  |
| High | Drop0 | -1.09 | 0.28 | -3.87 | 0.0001 |
|  | Drop1 | -2.76 | 3.00 | -0.92 | 0.3576 |
|  | Drop2 | -98.79 | 12556.96 | -0.01 | 0.9937 |
| Moderate | Drop0 | 0.51 | 0.68 | 0.74 | 0.4561 |
|  | Drop1 | -3.51 | 1.21 | -2.91 | 0.0037 |
|  | Drop2 | -1.19 | 0.92 | -1.29 | 0.1962 |
| High-Decreased | Drop0 | 0.16 | 4.46 | 0.04 | 0.9715 |
|  | Drop1 | 1.04 | 2.42 | 0.43 | 0.6663 |
|  | Drop2 | -1.68 | 2.70 | -0.62 | 0.5343 |
| **Sigma** |  | 0.31 | 0.01 | 35.05 | <0.0001 |
| **Group membership** | | | | | |
| Low | (%) | 39.67 | 5.20 | 7.63 | <0.0001 |
| Moderate | (%) | 55.03 | 5.11 | 10.77 | <0.0001 |
| High-Decreased | (%) | 5.30 | 1.49 | 3.56 | 0.0004 |

**Table S6: Trajectory model parameter estimates of negative symptoms in patients.**

| Trajectory | Parameter | Estimate | Standard Error | T-value | P-value |
| --- | --- | --- | --- | --- | --- |
| Group |  |  |  |  |  |
| Low | Intercept | 12.45 | 0.32 | 39.39 | <0.0001 |
|  | **Linear** | **-0.71** | **0.14** | **-4.99** | **<0.0001** |
| High-Decreased | Intercept | 44.89 | 2.33 | 19.24 | <0.0001 |
|  | Linear | -25.12 | 2.59 | -9.68 | <0.0001 |
|  | **Quadratic** | **4.81** | **0.63** | **7.61** | **<0.0001** |
| High-Increased | Intercept | 17.63 | 0.96 | 18.44 | <0.0001 |
|  | **Linear** | **1.94** | **0.48** | **4.02** | **0.0001** |
| Dropout |  |  |  |  |  |
| Low | Drop0 | -1.14 | 0.45 | -2.52 | 0.0119 |
|  | Drop1 | -0.01 | 0.03 | -0.17 | 0.8600 |
|  | Drop2 | 0.01 | 0.03 | 0.33 | 0.7414 |
| High-Decreased | Drop0 | -3.65 | 2.22 | -1.65 | 0.0993 |
|  | Drop1 | 0.05 | 0.08 | 0.57 | 0.5662 |
|  | Drop2 | 0.06 | 0.07 | 0.84 | 0.4032 |
| High-Increased | Drop0 | -4.50 | 1.87 | -2.41 | 0.0162 |
|  | Drop1 | 0.24 | 0.07 | 3.16 | 0.0016 |
|  | Drop2 | -0.07 | 0.06 | -1.26 | 0.2066 |
| Sigma |  | 4.19 | 0.07 | 58.92 | <0.0001 |
| Group membership | | | | | |
| Low | (%) | 69.54 | 2.09 | 33.22 | <0.0001 |
| High-Decreased | (%) | 16.35 | 2.10 | 7.77 | <0.0001 |
| High-Increased | (%) | 14.11 | 1.73 | 8.16 | <0.0001 |

**Table S7: Trajectory model parameter estimates of negative symptoms in siblings.**

| Trajectory | Parameter | Estimate | Standard Error | T-value | P-value |
| --- | --- | --- | --- | --- | --- |
| Groups |  |  |  |  |  |
| Low | Intercept | -0.01 | 0.04 | -0.25 | 0.7996 |
|  | **Linear** | **0.03** | **0.01** | **2.13** | **0.0334** |
| Moderate | **Intercept** | **0.30** | **0.02** | **15.47** | **<0.0001** |
| High-Increased | Intercept | -0.05 | 0.17 | -0.26 | 0.7927 |
|  | Linear | 0.56 | 0.19 | 2.83 | 0.0047 |
|  | **Quadratic** | **-0.10** | **0.05** | **-2.09** | **0.0366** |
| High-Decreased | Intercept | 1.57 | 0.21 | 7.50 | <0.0001 |
|  | Linear | -0.76 | 0.23 | -3.34 | 0.0008 |
|  | **Quadratic** | **0.16** | **0.05** | **2.95** | **0.0032** |
| Dropout |  |  |  |  |  |
| Low | Drop0 | -2.18 | 0.33 | -6.67 | <0.0001 |
|  | Drop1 | 5.36 | 1.99 | 2.69 | 0.0071 |
|  | Drop2 | 1.81 | 2.29 | 0.79 | 0.4291 |
| Moderate | Drop0 | -1.68 | 1.10 | -1.53 | 0.1261 |
|  | Drop1 | 0.34 | 1.80 | 0.19 | 0.8506 |
|  | Drop2 | -1.35 | 1.62 | -0.84 | 0.4031 |
| High-Increased | Drop0 | -2.55 | 2.28 | -1.12 | 0.2643 |
|  | Drop1 | 1.58 | 2.42 | 0.65 | 0.5131 |
|  | Drop2 | 0.52 | 2.62 | 0.20 | 0.8436 |
| High-Decreased | Drop0 | -1.61 | 2.55 | -0.63 | 0.5295 |
|  | Drop1 | -1.10 | 1.83 | -0.60 | 0.5480 |
|  | Drop2 | 1.16 | 2.28 | 0.51 | 0.6116 |
| Sigma |  | 0.20 | 0.004 | 49.52 | <0.0001 |
| Group membership | | | | | |
| Low | (%) | 35.15 | 5.72 | 6.15 | <0.0001 |
| Moderate | (%) | 51.70 | 4.93 | 10.50 | <0.0001 |
| High-Increased | (%) | 8.04 | 2.16 | 3.71 | <0.0001 |
| High-Decreased | (%) | 5.11 | 1.04 | 4.89 | <0.0001 |

**Table S8: Trajectory model parameter estimates of negative symptoms in controls.**

| Trajectory | Parameter | Estimate | Standard Error | T-value | P-value |
| --- | --- | --- | --- | --- | --- |
| Group |  |  |  |  |  |
| Low | **Intercept** | **-0.03** | **0.05** | **-0.49** | **0.6196** |
| Moderate | **Intercept** | **0.22** | **0.02** | **10.25** | **<0.0001** |
| High | **Intercept** | **0.58** | **0.03** | **21.98** | **<0.0001** |
| Dropout |  |  |  |  |  |
| Low | Drop0 | -1.34 | 0.50 | -2.69 | 0.0072 |
|  | Drop1 | 2.57 | 6.09 | 0.42 | 0.6724 |
|  | Drop2 | -1.31 | 12.30 | -0.11 | 0.9149 |
| Moderate | Drop0 | -0.98 | 0.83 | -1.17 | 0.2399 |
|  | Drop1 | -1.73 | 1.75 | -0.99 | 0.3239 |
|  | Drop2 | 0.47 | 1.37 | 0.35 | 0.7288 |
| High | Drop0 | -1.13 | 3.08 | -0.39 | 0.7131 |
|  | Drop1 | -0.32 | 3.06 | -0.11 | 0.9154 |
|  | Drop2 | -0.36 | 2.23 | -0.16 | 0.8711 |
| Sigma |  | 0.19 | 0.01 | 37.004 | <0.0001 |
| Group membership | | | | | |
| Low | (%) | 21.84 | 8.61 | 2.54 | 0.0113 |
| Moderate | (%) | 65.00 | 7.78 | 8.35 | <0.0001 |
| High | (%) | 13.16 | 2.45 | 5.38 | <0.0001 |

Table S9: Univariable multinomial random-effects logistic regression model on the environmental predictors of positive and negative symptoms trajectories in patients.

| Predictors | Positive symptom trajectories (Ref=Low) | | | | Negative symptom trajectories (Ref=Low) | | | |
| --- | --- | --- | --- | --- | --- | --- | --- | --- |
|  | **Moderate** | | **High** | | **High-Decreased** | | **High-Increased** | |
|  | **OR (95%CI)** | **P-value** | **OR (95%CI)** | **P-value** | **OR (95%CI)** | **P-value** | **OR (95%CI)** | **P-value** |
| PRS_SCZ_ |  |  |  |  |  |  |  |  |
| PRS (p<5x10^-8^) | 1.09 (0.80,1.37) | 0.53 | 1.28(0.76,1.80) | 0.24 | 1.14(0.78,1.50) | 0.41 | 0.97(0.63,1.31) | 0.87 |
| PRS (p<0.05) | 1.02(0.97,1.06) | 0.42 | 1.02(0.96,1.08) | 0.52 | 1.01(0.96,1.05) | 0.79 | 1.02(0.96,1.07) | 0.57 |
| PRS (p<0.1) | 1.02(0.97,1.06) | 0.40 | 1.03(0.98,1.09) | 0.25 | 1.02(0.97,1.06) | 0.43 | 1.01(0.96,1.06) | 0.64 |
| PRS (p<0.5) | 1.01(0.96,1.07) | 0.64 | 1.04(0.98,1.09) | 0.22 | 1.01(0.97,1.05) | 0.56 | 1.01(0.97,1.05) | 0.68 |
| PRS (p<1.0) | 1.01(0.95,1.06) | 0.73 | 1.03(0.97,1.08) | 0.26 | 1.01(0.97, 1.05) | 0.59 | 1.01(0.96,1.05) | 0.72 |
| Demographic and clinical characteristics | |  |  |  |  |  | |  |
| Age | 0.98(0.97,1.01) | 0.16 | 1.01(0.99,1.04) | 0.28 | 0.97(0.95,1.00) | 0.05 | 1.02(0.99,1.04) | 0.22 |
| Gender, female | 0.64(0.45,0.91) | 0.01 | 0.24(0.12,0.51) | 0.0002 | 0.54(0.32,0.92) | 0.023 | 0.60(0.34,1.05) | 0.07 |
| Marital status, married | 0.29(0.15,0.59) | 0.0006 | 0.16(0.04,0.68) | 0.013 | 0.61(0.29,1.29) | 0.19 | 0.60(0.27,1.36) | 0.22 |
| Ethnicity, non-Caucasian | 1.13(0.78,1.63) | 0.53 | 2.41(1.52,3.84) | 0.0002 | 1.15(0.70,1.87) | 0.58 | 1.54(0.94,2.53) | 0.09 |
| Years of education (full-time) | 0.97(0.93,1.01) | 0.13 | 0.97(0.92,1.03) | 0.36 | 0.93(0.89,0.98) | 0.006 | 0.96(0.91,1.02) | 0.15 |
| Age onset of first psychosis | 0.98(0.96,0.99) | 0.03 | 0.97(0.94,1.01) | 0.06 | 0.97(0.95,0.99) | 0.03 | 1.00(0.98,1.03) | 0.75 |
| Duration of illness | 1.02(0.98,1.07) | 0.30 | 1.09(1.03,1.15) | 0.001 | 0.99(0.95,1.03) | 0.64 | 1.02(0.98,1.06) | 0.29 |
| Number of psychotic episodes | 1.06(0.93,1.20) | 0.39 | 1.22(1.04,1.43) | 0.012 | 0.93(0.78,1.10) | 0.38 | 0.97(0.82,1.15) | 0.72 |
| Antipsychotics, first generation* | 1.78(1.04,3.05) | 0.035 | 1.76(0.84,3.71) | 0.14 | 1.62(0.81,3.21) | 0.17 | 1.60(0.77,3.32) | 0.21 |
| Cannabis use | 1.56(1.16,2.10) | 0.003 | 1.96(1.27,3.02) | 0.002 | 1.65(1.08,2.53) | 0.02 | 1.33(0.85,2.10) | 0.21 |
| Premorbid adjustment | 1.63(1.36,1.96) | <0.0001 | 1.68(1.28,2.19) | 0.0001 | 1.86(1.22,2.82) | 0.004 | 2.22(1.44,3.42) | 0.0003 |
| Estimated current IQ | 0.99(0.97,0.99) | 0.003 | 0.98(0.97,0.99) | 0.016 | 0.98(0.97,0.99) | 0.002 | 0.97(0.96,0.99) | 0.0004 |
| Depression^Ɨ^ | 1.20(1.13,1.27) | <0.0001 | 1.21(1.11,1.31) | <0.0001 | 1.06(0.95,1.19) | 0.30 | 1.22(1.09,1.36) | 0.0004 |
| General cognition | 0.87(0.78,0.96) | 0.009 | 0.80(0.69,0.92) | 0.002 | 0.79(0.69,0.90) | <0.0001 | 0.78(0.68,0.90) | <0.0001 |
| Psychotic symptoms | |  |  |  | |  | |  |
| Positive symptoms | 1.39(1.31,1.42) | <0.0001 | 2.09(1.88,2.33) | <0.0001 | 1.10(1.05,1.15) | <0.0001 | 1.08(1.04,1.14) | <0.0001 |
| Negative symptoms | 1.06(1.04,1.09) | <0.0001 | 1.12(1.08,1.15) | <0.0001 | 2.19(1.97,2.45) | <0.0001 | 1.54(1.43,1.65) | <0.0001 |
| Disorganization | 1.15(1.08,1.22) | <0.0001 | 1.28(1.19,1.38) | <0.0001 | 1.26(1.11,1.42) | <0.0001 | 1.22(1.08,1.38) | <0.0001 |
| Emotional distress | 1.23(1.09,1.38) | <0.0001 | 1.43(1.26,1.64) | <0.0001 | 1.15(1.09,1.22) | <0.0001 | 1.11(1.04,1.18) | <0.0001 |
| Excitement | 1.24(1.13,1.37) | <0.0001 | 1.46(1.31,1.63) | <0.0001 | 1.35(1.22,1.48) | <0.0001 | 1.24(1.13,1.36) | <0.0001 |
| Quality of life and functioning | |  |  |  | |  | |  |
| Quality of life | 0.34(0.22,0.55) | <0.0001 | 0.22(0.12,0.40) | <0.0001 | 0.26(0.12,0.55) | <0.0001 | 0.24(0.11,0.51) | <0.0001 |
| Social functioning^Ɨ^ | 0.93(0.92,0.95) | <0.0001 | 0.92(0.89,0.95) | <0.0001 | 0.90(0.84,0.97) | 0.007 | 0.86(0.79,0.93) | 0.0002 |
| Global functioning | 0.97(0.95,0.99) | 0.01 | 0.94(0.92,0.97) | 0.0001 | 0.94(0.91,0.97) | <0.0001 | 0.96(0.93,0.98) | <0.0001 |
| Occupational functioning^Ɨ^ | 0.86(0.82,0.91) | <0.0001 | 0.87(0.80,0.95) | <0.0001 | 0.92(0.85,1.00) | 0.06 | 0.84(0.77,0.92) | 0.0001 |
| Physical health *^Ɨ^* | |  |  |  | |  | |  |
| Body mass index (kg/m^2^) | 0.99(0.96,1.04) | 0.93 | 1.01(0.95,1.06) | 0.83 | 1.02(0.97,1.07) | 0.39 | 1.01(0.96,1.06) | 0.73 |
| Waist circumference (cm) | 1.00(0.99,1.02) | 0.52 | 1.01(0.99,1.03) | 0.44 | 1.01(0.99,1.03) | 0.21 | 1.01(0.99,1.03) | 0.22 |
| Glycated haemoglobin (mmol/mol) | 1.04(1.01,1.08) | 0.022 | 1.07(1.03,1.12) | 0.001 | 1.03(0.97,1.09) | 0.36 | 1.05(0.99,1.11) | 0.09 |
| Triglycerides (mmol/l) | 1.09(0.96,1.24) | 0.17 | 1.05(0.86,1.28) | 0.66 | 0.95(0.75,1.20) | 0.65 | 0.92(0.73,1.17) | 0.52 |
| High density lipoprotein (mmol/l) | 1.04(0.77,1.39) | 0.80 | 0.31(0.11,0.83) | 0.02 | 0.54(0.20,1.43) | 0.21 | 0.26(0.09,0.76) | 0.01 |
| Low density lipoprotein (mmol/l) | 1.06(0.85,1.32) | 0.60 | 1.55(1.13,2.11) | 0.006 | 1.01(0.71,1.44) | 0.97 | 0.87(0.61,1.24) | 0.45 |
| Diastolic blood pressure (mmHg) | 1.01(0.99,1.03) | 0.21 | 1.01(0.99,1.04) | 0.26 | 1.01(0.98,1.03) | 0.54 | 1.01(0.99,1.03) | 0.38 |
| Systolic blood pressure (mmHg) | 1.00(0.99,1.01) | 0.85 | 1.01(0.99,1.03) | 0.32 | 0.99(0.98,1.01) | 0.89 | 0.99(0.97,1.01) | 0.17 |
| Pulse rate (beat/min) | 1.01(1.01,1.03) | 0.022 | 1.03(1.01,1.04) | 0.004 | 1.02(0.99,1.03) | 0.11 | 1.01(0.99,1.03) | 0.16 |
| Metabolic syndrome score | 1.17(0.94,1.44) | 0.15 | 1.46(1.09,1.95) | 0.01 | 1.35(0.91,2.01) | 0.14 | 1.45(0.98,2.14) | 0.06 |

*^Ɨ^ = Used from the second wave at a three-year follow-up.*

**= Combination of medications and switching medication not considered, and the reference category is ‘second’ generation antipsychotics.*

Table S10: Univariable multinomial random-effects logistic regression model on the environmental predictors of positive and negative symptoms trajectories in siblings.

| Predictors | Positive symptom trajectories (Ref=Low) | | | | Negative symptom trajectories (Ref=Low) | | | |
| --- | --- | --- | --- | --- | --- | --- | --- | --- |
|  | **Moderate** | | **High (high and high-decreased)** | | **Moderate** | | **High (Increased and Decreased)** | |
|  | OR (95%CI) | P-value | OR (95%CI) | P-value | OR (95%CI) | P-value | OR (95%CI) | P-value |
| PRS_SCZ_ |  |  |  |  |  |  |  |  |
| PRS (p<5x10^-8^) | 1.03(0.66,1.38) | 0.89 | 1.20(0.72,1.68) | 0.38 | 1.21(0.87,1.55) | 0.18 | 1.05(0.61,1.50) | 0.81 |
| PRS (p<0.05) | 1.02(0.97,1.07) | 0.38 | 1.09(1.03,1.15) | 0.003 | 1.01(0.97,1.05) | 0.61 | 1.04(0.98,1.11) | 0.16 |
| PRS (p<0.1) | 1.02(0.97,1.06) | 0.37 | 1.07(1.02,1.13) | 0.006 | 1.01(0.98,1.05) | 0.51 | 1.05(0.99,1.10) | 0.07 |
| PRS (p<0.5) | 1.01(0.97,1.04) | 0.76 | 1.04(0.99,1.09) | 0.06 | 1.01(0.98,1.04) | 0.60 | 1.03(0.98,1.08) | 0.17 |
| PRS (p<1.0) | 1.00(0.97,1.04) | 0.84 | 1.04(0.99,1.08) | 0.08 | 1.00(0.97,1.03) | 0.78 | 1.03(0.98,1.07) | 0.21 |
| Demographic and clinical characteristics | |  | |  |  |  | |  |
| Age | 0.98(0.96,1.01) | 0.17 | 0.97(0.94,0.99) | 0.016 | 1.00(0.98,1.02) | 0.97 | 1.01(0.99,1.04) | 0.29 |
| Gender, female | 1.62(1.12,2.32) | 0.01 | 1.77(1.15,2.71) | 0.01 | 1.27(0.94,1.73) | 0.12 | 0.75(0.47,1.19) | 0.22 |
| Marital status, married | 1.02(0.71,1.48) | 0.91 | 0.63(0.40,0.98) | 0.04 | 0.90(0.66,1.24) | 0.53 | 0.45(0.27,0.76) | 0.003 |
| Ethnicity, non-Caucasian | 2.46(1.37,4.42) | 0.003 | 2.38(1.23,4.58) | 0.01 | 1.08(0.70,1.67) | 0.73 | 1.18(0.63,2.21) | 0.61 |
| Years of education (full-time) | 0.94(0.89,0.99) | 0.013 | 0.92(0.87,0.98) | 0.008 | 0.99(0.95,1.03) | 0.67 | 0.95(0.90,1.01) | 0.10 |
| Cannabis use | 1.75(1.04,2.96) | 0.035 | 2.44(1.37,4.36) | 0.002 | 1.54(0.99,2.40) | 0.06 | 1.99(1.09,3.61) | 0.02 |
| Premorbid adjustment | 1.70(1.22,2.36) | 0.002 | 2.49(1.72,3.59) | <0.0001 | 2.78(1.98,3.92) | <0.0001 | 7.13(4.59,11.08) | <0.0001 |
| Estimated current IQ | 0.99(0.98,1.01) | 0.19 | 0.98(0.97,0.99) | 0.02 | 0.99(0.98,1.01) | 0.46 | 0.99(0.98,1.01) | 0.48 |
| Depression^Ɨ^ | 1.01(0.52,1.97) | 0.97 | 1.49(0.78,2.85) | 0.23 | 0.99(0.68,1.45) | 0.98 | 1.43(0.99,2.07) | 0.06 |
| General cognition | 1.01(0.88,1.16) | 0.87 | 0.95(0.82,1.10) | 0.48 | 0.97(0.87,1.07) | 0.55 | 0.93(0.80,1.07) | 0.31 |
| Psychotic-like experiences (distress) | |  | |  |  |  | |  |
| Positive symptoms | 1.92(1.08,3.39) | 0.026 | 6.58(3.56,12.15) | <0.0001 | 2.95(1.76,4.95) | <0.0001 | 9.98(5.32,18.70) | <0.0001 |
| Negative symptoms | 3.04(1.77,5.24) | <0.0001 | 14.04(7.65,25.77) | <0.0001 | 2.49(1.65,3.76) | <0.0001 | 8.48(4.98,14.46) | <0.0001 |
| Depressive symptoms | 1.96(1.29,2.99) | 0.002 | 7.21(4.44,11.70) | <0.0001 | 2.23(1.54,3.23) | <0.0001 | 6.93(4.27,11.25) | <0.0001 |
| Quality of life and functioning | |  | |  |  |  | |  |
| Quality of life | 0.28(0.17,0.47) | <0.0001 | 0.06(0.04,0.12) | <0.0001 | 0.15(0.09,0.26) | <0.0001 | 0.02(0.01,0.05) | <0.0001 |
| Social functioning^Ɨ^ | 0.98(0.94,1.02) | 0.30 | 0.93(0.89,0.97) | 0.0004 | 0.91(0.87,0.95) | <0.0001 | 0.78(0.74,0.83) | <0.0001 |
| Occupational functioning^Ɨ^ | 0.85(0.74,0.97) | 0.019 | 0.76(0.66,0.88) | 0.002 | 0.87(0.79,0.97) | 0.009 | 0.76(0.68,0.85) | 0.0001 |

*^Ɨ^ = Used from the second wave at a three-year follow-up.*

Table S11: Univariable multinomial random-effects logistic regression model on the environmental predictors of positive and negative symptoms trajectories in controls.

| Predictors | Positive symptom trajectories (Ref=Low) | | | | Negative symptom trajectories (Ref=Low) | | | |
| --- | --- | --- | --- | --- | --- | --- | --- | --- |
|  | **Moderate** | | **High-Decreased** | | **Moderate** | | **High** | |
|  | **OR (95%CI)** | **P-value** | **OR (95%CI)** | **P-value** | **OR (95%CI)** | **P-value** | **OR (95%CI)** | **P-value** |
| PRS_SCZ_ |  |  |  |  |  |  |  |  |
| PRS (p<5x10^-8^) | 2.12(0.55,3.69) | 0.046 | 2.61(0.08,5.13) | 0.05 | 1.17(0.72,1.62) | 0.42 | 1.02(0.49,1.55) | 0.94 |
| PRS (p<0.05) | 1.03(0.94,1.12) | 0.50 | 0.97(0.84,1.09) | 0.62 | 1.01(0.95,1.07) | 0.67 | 0.99(0.92,1.07) | 0.98 |
| PRS (p<0.1) | 1.04(0.96,1.12) | 0.34 | 0.97(0.86,1.07) | 0.57 | 1.01(0.96,1.06) | 0.61 | 1.01(0.94,1.07) | 0.82 |
| PRS (p<0.5) | 1.04(0.96,1.11) | 0.32 | 0.98(0.89,1.07) | 0.69 | 1.02(0.97,1.06) | 0.44 | 1.02(0.96,1.07) | 0.58 |
| PRS (p<1.0) | 1.04(0.96,1.11) | 0.32 | 0.98(0.88,1.07) | 0.64 | 1.02(0.97,1.06) | 0.40 | 1.01(0.96,1.07) | 0.61 |
| Demographic and clinical characteristics | |  | |  |  |  | |  |
| Age | 0.97(0.94,1.01) | 0.09 | 0.94(0.89,0.99) | 0.018 | 0.99(0.97,1.02) | 0.85 | 1.01(0.98,1.04) | 0.62 |
| Gender, female | 1.15(0.64,2.05) | 0.64 | 2.63(0.95,7.32) | 0.06 | 1.09(0.68,1.75) | 0.73 | 1.24(0.65,2.35) | 0.51 |
| Marital status, married | 0.81(0.44,1.51) | 0.51 | 0.75(0.28,2.04) | 0.58 | 1.32(0.79,2.20) | 0.28 | 0.79(0.39,1.58) | 0.50 |
| Ethnicity, non-Caucasian | 4.47(1.22,16.40) | 0.024 | 3.33(0.49,22.52) | 0.22 | 2.17(0.76,6.17) | 0.14 | 1.32(0.32,5.41) | 0.70 |
| Years of education (full-time) | 0.93(0.85,1.02) | 0.11 | 0.93(0.80,1.08) | 0.33 | 1.03(0.96,1.12) | 0.39 | 1.03(0.93,1.13) | 0.60 |
| Cannabis use | 2.82(1.03,7.61) | 0.04 | 6.71(1.89,23,82) | 0.003 | 0.92(0.46,1.83) | 0.81 | 0.70(0.27,1.84) | 0.47 |
| Premorbid adjustment | 2.40(1.30,4.44) | 0.005 | 3.83(1.67,8.80) | 0.002 | 3.31(1.52,7.18) | 0.003 | 10.08(4.28,23.72) | <0.0001 |
| Estimated current IQ | 0.98(0.96,0.99) | 0.046 | 0.97(0.94,1.01) | 0.05 | 1.01(0.99,1.02) | 0.66 | 0.99(0.97,1.02) | 0.68 |
| Depression^Ɨ^ | 1.06(0.66,1.69) | 0.82 | 1.13(0.62,2.06) | 0.69 | 3.23(0.47,22.38) | 0.23 | 3.90(0.56,27.39) | 0.17 |
| General cognition | 0.90(0.73,1.09) | 0.28 | 0.84(0.63,1.14) | 0.26 | 1.09(0.95,1.26) | 0.21 | 0.91(0.76,1.10) | 0.34 |
| Psychotic-like experiences (distress) | |  | |  | |  | |  |
| Positive symptoms | 4.02(1.53,10.54) | 0.005 | 10.78(3.25,35.76) | 0.0001 | 4.80(1.55,14.88) | 0.007 | 18.11(5.27,62.23) | <0.0001 |
| Negative symptoms | 3.54(1.63,7.72) | 0.002 | 13.46(4.49,40.35) | <0.0001 | 4.91(1.54,15.67) | 0.007 | 13.78(3.96,48.00) | <0.0001 |
| Depressive symptoms | 2.98(1.53,5.80) | 0.001 | 10.50(4.21,26.20) | <0.0001 | 3.57(1.74,7.32) | 0.0005 | 8.80(3.85,20.10) | <0.0001 |
| Quality of life and functioning | |  | |  | |  | |  |
| Quality of life | 0.07(0.02,0.27) | 0.0001 | 0.02(0.01,0.07) | <0.0001 | 0.11(0.03,0.40) | 0.0008 | 0.02(0.01,0.06) | <0.0001 |
| Social functioning^Ɨ^ | 0.66(0.51,0.85) | 0.001 | 0.64(0.49,0.83) | 0.002 | 0.89(0.83,0.96) | 0.002 | 0.80(0.73,0.87) | <0.0001 |
| Occupational functioning^Ɨ^ | 0.82(0.69,0.96) | 0.016 | 0.73(0.59,0.90) | 0.003 | 0.88(0.76,1.03) | 0.11 | 0.85(0.71,1.01) | 0.07 |

*^Ɨ^ = Used from the second wave at a three-year follow-up.*

**References**

1. Jones BL, Nagin DS, Roeder K. A SAS procedure based on mixture models for estimating developmental trajectories. *Sociol Methods Res*. 2001;29(3):374-393.

2. Haviland AM, Jones BL, Nagin DS. Group-based trajectory modeling extended to account for nonrandom participant attrition. *Sociological Methods & Research*. 2011;40(2):367-390.

3. Dong Y, Peng CJ. Principled missing data methods for researchers. *SpringerPlus*. 2013;2(1):222.

4. Levine SZ, Rabinowitz J. Trajectories and antecedents of treatment response over time in early-episode psychosis. *Schizophr Bull*. 2010;36(3):624-632.

5. Niyonkuru C, Wagner AK, Ozawa H, Amin K, Goyal A, Fabio A. Group-based trajectory analysis applications for prognostic biomarker model development in severe TBI: A practical example. *J Neurotrauma*. 2013;30(11):938-945.

6. Nagin DS. Analyzing developmental trajectories: A semiparametric, group-based approach. *Psychol Methods*. 1999;4(2):139-157.

7. Nagin DS, Odgers CL. Group-based trajectory modeling in clinical research. *Annu Rev Clin Psychol*. 2010;6:109-138.

8. Nagin DS. Group-based trajectory modeling: An overview. *Ann Nutr Metab*. 2010;65(2-3):205-210.

9. Purcell S, Neale B, Todd-Brown K, et al. PLINK: A tool set for whole-genome association and population-based linkage analyses. *Am J Hum Genet*. 2007;81(3):559-575.

10. Das S, Forer L, Schönherr S, et al. Next-generation genotype imputation service and methods. *Nat Genet*. 2016;48(10):1284.

11. Ripke S, Neale BM, Corvin A, et al. Biological insights from 108 schizophrenia-associated genetic loci. *Nature*. 2014;511(7510):421-7.

12. Andruff H, Carraro N, Thompson A, Gaudreau P, Louvet B. Latent class growth modelling: A tutorial. *Tutor Quant Methods Psychol*. 2009;5(1):11-24.
